# British Medicinal Plants

**Published:** 1891-12

**Authors:** Alfred Heath

**Affiliations:** London, England


### BRITISH MEDICINAL PLANTS.

ALFRED HEATH, M. D., F. L. S., LONDON, ENGLAND.

### ORDER 33.—PARONYCHIACEÆ.

*Herniaria glabra* (Rupture wort).—This plant was at one time famed as a cure for hernia, as its name implies. It is anti-venereal. The internal as well as the external use of rupture-wort is said to cure rupture in both children and grown-up people. It has been successfully used against gonorrhœa, strangury, stone and gravel pains in abdomen, obstructions of the liver, in jaundice, worms, etc. It is a famed wound-wort and cleanses and promotes healing of fistulous ulcers. It has not been used much in homœopathic medicine, but has been successfully given in acute and chronic cystitis. It is said to abort gonorrhœa by preventing the implication of the deeper parts of the urethra.

### ORDER 34.—CRASSULACEÆ.

*Sedum teléphium* (Orpine, Live-long).—This plant is a wound-wort of the first order, and has been used with considerable success in the treatment of wounds and ulcers, both internal and external, such as ulcers in the lungs, liver, womb, etc., hemorrhages from various parts of the body, especially from the bowels. It is good for piles with great soreness of the rectum and relieves the burning and heat. Applied externally to wounds, it eases the pain and promotes healing. A bruised leaf applied to a fresh wound quickly heals it. It is also good for burns and bruises and it is a powerful diuretic.

*Sedum acre* (Stone-crop, wall pepper).—This plant is also a famed wound-wort and a powerful styptic for both internal and external wounds. It heals fretting sores and ulcers, removes heated conditions of the body. It is good in fevers of various kinds, agues, etc. It has been found useful in scrofulous conditions as an application, but must be carefully used or it will blister the skin. It is good for soreness of the mouth. The juice taken internally will excite vomiting.

*Sempervivum tectorum* (The House-leek).—This plant very commonly grows on the roofs of cottages in the villages. There is a curious superstition respecting it that it preserves from fire and lightning the place it grows upon. It is very similar in its action to the two last-mentioned plants. It is useful in burning heats of the eyes and other parts, in fevers, agues, thirst, etc. It lessens excessive menstrual discharges. It has been found useful in erysepelas, scaldings and burnings, for the shingles, fretting ulcers, ring-worms, etc. It relieves the pain of gout. The juice is said to remove warts and corns from the hands and feet. It cools the head and stops bleeding of the nose very quickly. The leaves rubbed on places stung with nettles or bees quickly takes away the pain.

*Cotyledon umbilicus* (Navel-wort, Penny pies).—The action of this plant is similar to the House-leek and Sedums. It is cooling in fever and in liver diseases, and it is also diuretic. The juice made into an ointment has been used with success to relieve the pain of erysipelas, shingles, piles, chillblains, etc. There is a proving in Allen's *Materia Medica*.

ORDER 37.—UMBELLIFERÆ.

*Eryngium maritimum* (Sea-Holly).—Very little used in medicine, but some mention has been made of it in homœopathic literature. It is a lovely plant common to our sea-shores. The leaves are very thorny, but of a deep blue, as also is some part of the stem, the flowers also are blue. The wort is the part used. It is very mucilaginous and decoctions are used in chest troubles. It is said to be aphrodisiac.

